# Letter to the Editor: Reconsidering the Clinical Role of the Prognostic Nutritional Index in Cancer

**DOI:** 10.1016/j.advnut.2026.100672

**Published:** 2026-06-09

**Authors:** Longying Zhang, Lifen Zhao, Lina Liu

**Affiliations:** Second Hospital of Dalian Medical University, Dalian, China

Dear Editor:

We read with interest the umbrella review by Li et al. [1] entitled “Prognostic nutritional index and cancer prognostic outcomes: an umbrella review of systematic reviews and meta-analyses of observational studies.” The authors synthesized 64 meta-analyses and reported that higher prognostic nutritional index (PNI) was consistently associated with improved overall survival across multiple malignancies, with 84.5% of quantitative syntheses reaching statistical significance. While this body of evidence appears coherent, its clinical interpretation requires closer scrutiny.

A key issue is whether the observed association between PNI and survival reflects an independent prognostic signal or merely recapitulates baseline disease severity and treatment tolerance. PNI is constructed from serum albumin and lymphocyte count, both of which are sensitive to systemic inflammation, tumor burden, and comorbid conditions. In advanced cancer, hypoalbuminemia is frequently driven by cachexia and cytokine-mediated catabolism rather than nutritional intake alone, and lymphopenia is a well-established marker of impaired host immunity and disease progression [2,3]. In this context, a low PNI may reflect aggressive disease biology rather than precede it. The consistent hazard ratios reported across cancer types may therefore reflect a shared pathway of systemic deterioration rather than a modifiable or independently informative prognostic factor.

This distinction has direct clinical consequences. Prognostic factors that are strongly associated with survival do not necessarily improve risk stratification if they overlap with variables already used in clinical decision making. Tumor stage, performance status, and treatment method remain dominant determinants of outcome. Without demonstrating incremental predictive value beyond these variables, the addition of PNI risks duplicating existing information. In prognostic research, improvement in discrimination or reclassification is often modest even for statistically significant markers [4]. The present review does not address whether PNI meaningfully improves model performance or alters clinical categorization, which limits its immediate applicability at the bedside.

A related concern is the interpretation of PNI as a target for intervention. Although it is tempting to infer that improving PNI may translate into better outcomes, this assumption is not supported by the evidence synthesized in the review. Interventions that raise serum albumin or lymphocyte count do not necessarily reverse the underlying inflammatory or metabolic drivers of cancer progression. Trials of nutritional supplementation in oncology have shown mixed effects on survival despite improvements in nutritional parameters [5]. Therefore, PNI may function more as a marker of physiological reserve than as a modifiable determinant of prognosis.

Taken together, the findings by Li et al. [1] support the consistency of the association between PNI and cancer outcomes, but they do not yet establish a role for PNI in clinical decision making. The distinction between association, stratification, and actionability is essential. Without evidence that PNI changes how patients are classified or managed, its value remains descriptive rather than directive.

## Author contributions

The authors’ responsibilities were as follows—All authors wrote the original draft, reviewed the content, and read and approved the final manuscript.

## Declaration of Generative AI and AI-assisted technologies in the writing process

The authors declare that no generative AI or AI-assisted technologies were used in the writing of this manuscript.

## Funding

The authors reported no funding received for this study.

## Conflict of interest

The authors report no conflicts of interest.
